# Validation of a Drug-Related Problem Classification System for the Intermediate and Long-Term Care Setting in Singapore

**DOI:** 10.3390/pharmacy6040109

**Published:** 2018-10-03

**Authors:** Xin Yan Lim, Quan Qi Yeo, Grace Li Lin Kng, Wing Lam Chung, Kai Zhen Yap

**Affiliations:** 1 Pharmacy Department, Mount Alvernia Hospital, Singapore 574623, Singapore; lim.xin.yan@mtalvernia.sg; 2 Watson’s Personal Care Stores Pte Ltd., Singapore 079907, Singapore; quanqi.yeo@watsons.com.sg (Q.Q.Y.); grace.lin@watsons.com.sg (G.L.L.K.); wl.chung@watsons.com.sg (W.L.C.); 3 Pharmaceutical Society of Singapore (PSS) Intermediate and Long-Term Care (ILTC) Pharmacists Workgroup, Singapore 159457, Singapore; 4 Department of Pharmacy, Faculty of Science, National University of Singapore, Singapore 117543, Singapore

**Keywords:** drug-related problem, intermediate and long-term care, classification system, validation, nursing home

## Abstract

**Background**: This study aims to evaluate the inter-rater reliability and perceived usability of a newly developed drug-related problem (DRP) classification system for use by pharmacists in the intermediate and long-term care (ILTC) setting in Singapore. Methods: This was a cross-sectional survey study involving the use of a self-administered questionnaire. All 55 pharmacists affiliated to the Pharmaceutical Society of Singapore (PSS) ILTC Pharmacists Workgroup who were above 21 years old and not authors of the classification system were invited to participate. The inter-rater reliability of participants’ classification of 46 mock DRP cases using the new DRP classification system was determined using Fleiss’s kappa (κ). Participants’ perceived usability of the classification system was evaluated using six items with five-point Likert scales (1—“strongly disagree”, 5—“strongly agree”). **Results**: Thirty-three pharmacists responded to the survey. Overall inter-rater reliability was found to be substantial (κ = 0.614; 95% CI: 0.611–0.617). All usability items received positive ratings (“strongly agree” or “agree”) from at least 69% of participants. **Conclusion**: The new DRP classification system has substantial external validity and appears to be suitable for use by pharmacists to document and report DRPs in the ILTC setting in Singapore and facilitate evaluation of the impact of pharmaceutical care in the ILTC setting.

## 1. Introduction

A drug-related problem (DRP) is defined by the Pharmaceutical Care Network Europe (PCNE) as an event involving drug therapy that actually or potentially interferes with desired health outcomes [1]. DRPs are undesirable and often lead to increased cost, morbidity, and mortality [2]. Although pharmacists are known as the lead healthcare professional in identifying, resolving, and preventing DRPs [3], evaluations of the quality and value of such pharmacist interventions continue to be pivotal in the development of new pharmaceutical care services and for increasing knowledge about the types and frequencies of DRPs observed in different clinical settings [4]. However, such studies cannot be feasible without the use of a standardized documentation and classification system by pharmacists for documenting and reporting DRPs [5,6,7,8].

In a review study on the application of DRP classification systems [9], Basger et al. found 20 different types of classification systems being used in their unmodified or modified forms among the 268 original studies included in their review. Among these, the most commonly used classification systems were from Hepler and Strand [10], followed by Cipolle et al. [3] and PCNE. In their review, the most commonly reported reason for the choice of classification system used was related to the chosen classification system’s distribution and use in other studies. However, the likely reason for using a modified version of the selected classification system was to ensure that all identified DRPs could be classified. While modified DRP classification systems were used in many studies conducted at various care settings, it was found to be the most prevalent in studies at aged-care facilities (86%) [9]. These suggests the complexity of DRPs identified and the importance of using a classification system that can suit the needs of various practice settings.

The issue of DRPs is becoming increasingly important as Singapore faces an ageing population with increasing prevalence of chronic diseases and demand for intermediate and long-term care (ILTC) residential facilities, such as nursing homes [11]. There is a need to establish pharmaceutical services to ensure the quality and safety of medicine use in these care settings. For this purpose, a Pharmaceutical Care Programme for nursing homes was started by the Pharmaceutical Society of Singapore (PSS) and the Agency for Integrated Care in 2011. A PSS Nursing Home Pharmacists Workgroup was also formed at the same time to facilitate the sharing of practices by nursing home pharmacists so as to maintain the standards and quality of pharmaceutical services provided. Recently, this workgroup was renamed as the PSS ILTC Pharmacists Workgroup to include the growing pool of pharmacists serving community hospitals and hospices [12]. Due to this change, it is timely and of interest for the workgroup to evaluate the impact of pharmaceutical care in the ILTC setting in Singapore. However, the different DRP classification systems used by pharmacists at different ILTC institutions posed a challenge for the collation and analysis of data in such evaluation studies. 

Similar to the findings by Basger et al. [9], most of the DRP classification systems used by different local ILTC institutions were various modified versions of that by Hepler and Strand. Often, the modifications involved the addition of categories to capture transcribing errors and illegibility/ambiguity in drug orders. In some institutions, the DRP classification systems were modified to fit into the workflow and practices for routine clinical use in order to flag out nonadherence to institutional protocols. In addition, the lack of descriptors to allow accurate interpretation of the meaning of the DRP categories in the various versions of classification systems needs to be addressed before DRP data can be pooled for use in multi-institutional evaluation studies. Although existing classification by PCNE contains explicit definitions for its categories and appears to be the most comprehensive in its ability to capture the problem, causes, planned intervention, acceptance of intervention, and status of the DRP, many practitioners found it to be time consuming and cumbersome for use in their daily practice. 

Hence, a new DRP classification system specific for use in ILTC institutions in Singapore was developed by the PSS ILTC Pharmacists Workgroup. The development process involved consolidating a list of DRP categories based on those obtained from the various ILTC institutions in Singapore, with reference to the Hepler and Strand [10] and PCNE V7.0 categories. This list was then modified to better suit the local context and the ILTC setting using two rounds of consensus discussion held during the workgroup meetings involving all members. The resulting DRP classification consists of 8 main categories with a total of 24 subcategories, as shown in Table 1. 

A good DRP classification system should have an open hierarchical structure with clear definitions for each DRP category in order to reduce ambiguity in the coding of a problem (i.e., lead to one choice of coding for each problem). In addition, coding of the underlying cause of the DRP should be separate from the coding of the problem [13]. Validation has also been specified as being necessary and it is essential to validate this new DRP classification system prior to its launch. The inter-rater reliability of the classification system can reflect external validity [4]. Ideally, different users should select the same classification subcategory when presented with the same DRP case. Ease of use has also been specified as a requirement, and a good classification system should have acceptable usability [13].

The objective of the study was, therefore, to determine the external validity [4] in terms of inter-rater reliability of the new DRP classification system developed by the PSS ILTC Pharmacists Workgroup (by measuring inter-rater reliability) and evaluate the usability of the classification system as perceived by pharmacists.

## 2. Methods

### 2.1. Study Design and Participant Recruitment

This was a cross-sectional questionnaire study conducted from November to December 2016 that was extended to all pharmacists affiliated with the PSS ILTC Pharmacists Workgroup who were above 21 years old and not authors of the classification system. After obtaining ethics approval for this study from NUS IRB, the recruitment was conducted in two parts. The first recruitment was held during the workgroup meeting in November 2016, where a verbal invitation was extended to the pharmacists present, along with the provision of the link to the online self-administered version of the questionnaire in their meeting notes (as well as a hardcopy version of the questionnaire with an attached return envelope if they preferred). The second recruitment was done through representatives who helped to disseminate email invitations and reminders (along with the link to the online self-administered version of the questionnaire) to all affiliated ILTC pharmacists in their institution. A total of 55 pharmacists were reached to participate in the study. 

### 2.2. Data Collection

The first part of the questionnaire required participants to classify 46 mock patient cases containing DRP using the newly developed DRP classification. All the cases used were modified from real scenarios from nursing homes, which were selected (by CWL, KLLG, and YQQ) to cover all the subcategories of the new DRP classification system except for “others”. All 46 cases were also piloted and revised (by LXY, YKZ, CWL, KLLG, and YQQ) to ensure clarity, so as to avoid inconsistencies in inter-rater agreement measures [4]. 

In the second part of the questionnaire, participants were asked to rate six items pertaining to their perceived effectiveness, efficiency, and satisfaction of use for the classification system on five-point Likert scales (1—“strongly disagree”, 2—“disagree”, 3—“neutral”, 4—“agree”, and 5—“strongly agree”). These items were adapted from questionnaires used in other studies [14,15,16]. and piloted to ensure clarity before they were included in this survey. At the end of the questionnaire, participants were also asked to provide additional comments on how the classification system could be improved. 

### 2.3. Outcome Measures and Data Analysis

Inter-rater reliability of how the participants classified the 46 mock DRP cases was estimated using Fleiss’s kappa, which measures the degree of agreement between three or more raters rating subjects on a nominal scale corrected for chance [17]. This statistical test was performed on SPSS using an SPSS extension bundle for Fleiss’s kappa [18] with subanalyses of kappa values to identify if there were differences in agreement among individuals with different years of practice and ILTC experiences. Results were interpreted according to Landis and Koch [19], with a kappa value of 0.81–1.00 considered as “almost perfect”, 0.61–0.80 as “substantial”, 0.41–0.60 as “moderate”, 0.21–0.40 as “fair”, and 0.00–0.20 as “slight” agreement. A value of 0.60 was set as an acceptable level of agreement.

Participants’ perceived usability of the classification system was reported using the percentage of participants with positive (“agree” or “strongly agree”), neutral, and negative ratings (“disagree” or “strongly disagree”) for each question. In this study, acceptable usability was defined as having at least 60% response in positive ratings.

## 3. Results

### 3.1. Participants’ Demographics

Of the 55 pharmacists reached, 33 returned fully completed questionnaires, providing a 60% response rate. The participants’ demographics are summarised in Table 2. 

### 3.2. Inter-Rater Reliability

The overall inter-rater agreement among 33 participants’ classification of all 46 mock DRP cases using the newly developed DRP classification system was found to be substantial, with κ = 0.614 (95% CI: 0.611–0.617).

**Figure 1:**
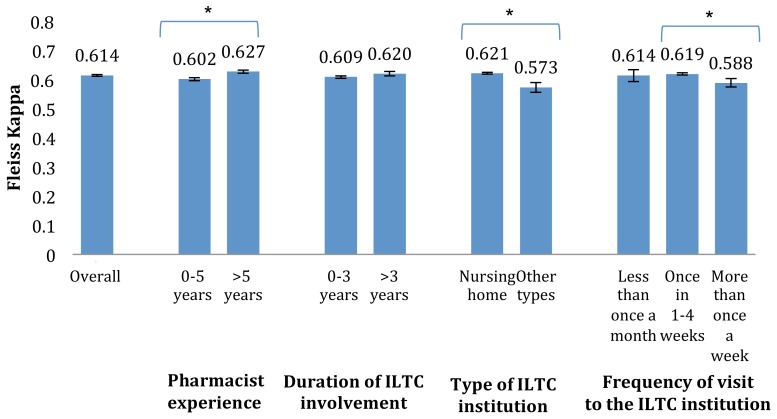
Kappa values for participants of various practice experiences (with 95% CI; * denotes significant difference).

Subtle differences in the inter-rater agreement were found between pharmacists with different work experiences (Figure 1). The inter-rater agreement among pharmacists with more than 5 years of experience as a pharmacist (κ = 0.627 with 95% CI: 0.621–0.633) was significantly greater than those with 5 or less years of experience (κ = 0.602 with 95% CI: 0.596–0.607). A similar trend was observed among pharmacists involved in nursing homes (κ = 0.621 with 95% CI: 0.618–0.624) in contrast to pharmacists involved in other ITLC institutions (κ = 0.573 with 95% CI: 0.556–0.590). 

No linear relationship was observed between the frequency of pharmacist visits to the ILTC institutions and the inter-rater agreement. However, the inter-rater agreement among pharmacists who visited the ILTC institution once every 1–4 weeks (κ = 0.619 with 95% CI: 0.615–0.623) was significantly higher than those who visited more than once a week (κ = 0.588 with 95% CI: 0.574–0.603). 

Although a higher inter-rater agreement was observed among pharmacists with more than 3 years of working experience in the ILTC setting compared to those with 3 or less years of working experience, this finding was not statistically significant (κ = 0.620 with 95% CI: 0.612–0.627 as compared to κ = 0.609 with 95% CI: 0.604–0.613).

### 3.3. Users’ Perceived Usability

The participants’ self-reported responses on the DRP classification system’s usability are reported in Table 3. No participants responded with “strongly disagree” for any question. Although participants had a generally positive opinion on the usability of the classification system with positive ratings (“strongly agree” or “agree”) of above 69% for all six items, four participants (12.1%) commented that there were too many subcategories in the classification system.

## 4. Discussion

### 4.1. Inter-Rater Reliability 

The DRP classification system developed by the PSS ILTC Pharmacists Workgroup achieved a substantial level of inter-rater agreement among the 33 pharmacists practicing in the ILTC setting. The Fleiss’s kappa value achieved in the study is acceptable and comparable to that of other classification systems reported in the literature, including the GSASA V2 [16] (k = 0.52), DOCUMENT [7] (k = 0.53), and APS-Doc [6] (κ = 0.68 for main categories and κ = 0.58 for subcategories). This demonstrated that the newly developed classification system can be a reliable tool for use in future studies for categorizing and collating a large number of real DRP cases by a large group of pharmacists in actual practice setting. Considering that the pharmacists did not undergo prior training on the use of the classification system before undertaking the survey, training sessions may improve the kappa score further. 

It should be noted, however, that the level of agreement among the pharmacists can be affected by practice experience and type of ILTC institution in which the pharmacist is involved [13]. As shown in the subanalysis of this study, pharmacists with practice experience of more than 5 years had significantly higher inter-rater agreement than those with lesser practice experience. 

In addition, the inter-rater agreement among pharmacists involved in nursing homes was significantly higher than those involved in other types of residential ILTC institutions. This could be because pharmacists involved in nursing homes were more familiar with the scenarios described in the 46 cases, which were all from nursing homes. Furthermore, different types of ILTC institutions can have different standard operating procedures, training, and commonly observed DRPs, which can influence the pharmacist’s classification choice. Hence, one should be aware of such potential differences and conduct subanalyses in future studies that are based on data collected using the classification system from various ILTC institutions.

There was no linear trend between the frequency of pharmacist visit to the ILTC institution and the inter-rater agreement among the pharmacists. Interestingly, pharmacists who visited the ILTC institution most frequently (more than once a week) appeared to have the lowest inter-rater agreement. Upon further analysis, it was found that four of the seven participants in this group were practicing in ILTC institutions other than nursing homes. The lower inter-rater agreement achieved could thus be attributed to their familiarity with the given mock cases used in this study instead of the frequency of visit. Furthermore, similar inter-rater agreement was found for the other two groups (once in 1–4 weeks and less once in a month). This suggests that that frequency of visits to the ILTC institution does not affect inter-rater agreement among pharmacists.

Though slight differences in the level of agreement were observed for different subgroups, the lowest kappa value obtained was 0.57, which is comparable to the overall agreement reported in other studies. Thus, the newly developed DRP classification system is suitable to be used as a standardized framework for documenting and reporting DRPs in the ILTC setting in Singapore. 

### 4.2. Users’ Perception of Usability 

The majority of participants had positive ratings for the usability of the classification system. This shows that they felt that the effectiveness and efficiency of the classification system was acceptable and they were satisfied with it. The positive perception of usability could be related to the pharmacists’ participation in the two-round consensus meeting during the development process, where their comments and inputs were incorporated in the final version of the classification system.

In contrast to PCNE V7.0, which allows the user to classify the DRP into the problem, causes, planned intervention, acceptance of intervention, and status of the DRP, the newly developed classification system only includes classification of the main problem. Although coding of the causes and interventions of DRPs can facilitate data analysis in research of pharmaceutical care impact, it can be complicated to use, time consuming [6], and impractical for routine use in clinical practice. Hence, a complex classification system can be counterproductive should pharmacists report a lack of willingness to use it in their practice. In any case, the causes, suggested interventions, and their outcomes would be documented along with the classification of the DRP as part of good practice in pharmaceutical care [3], which can subsequently be extracted for coding and analysis for research purposes. 

Furthermore, even though the new classification system only includes classification of the main problem and has 24 subcategories, which is similar to the DOCUMENT system (25–31 subcategories depending on version used) [7] and fewer than APS-Docs [6] (48 subcategories), there were still comments that there were too many subcategories. This highlights the difficulty in developing a classification system that can increase the ease of research and yet be simple and practical for use by practitioners. 

### 4.3. Limitations and Future Work

As the model cases were selected to cover the subcategories included in the classification system, they may not fully reflect the whole scope of DRPs seen in clinical practice. Hence, the completeness of categories in the classification system cannot be evaluated. 

Moving forward, the classification system should be piloted for use in various ILTC institutions to evaluate the practicality of the classification system in actual clinical practice. In addition, the internal validity of the classification system can be further investigated by evaluating its test–retest reliability, along with its ability to capture and report the complete and unique actual DRPs seen in clinical practice [14].

## 5. Conclusions

This study evaluated the external validity of the newly developed DRP classification system in terms of inter-rater reliability. As shown in the results, there was substantial agreement among the 33 pharmacists involved in the study of the 46 mock cases used. Users’ opinion on its usability was also positive in general. Therefore, this DRP classification system appears to be suitable and reliable for use by pharmacists to document and report DRPs in the ILTC setting in Singapore, which can in turn support future studies to evaluate the impact of pharmaceutical care in the ILTC setting and increase knowledge about the types and frequencies of DRPs observed in various ILTC institutions.

## Figures and Tables

**Table 1:** Summary of the main and subcategories of the Pharmaceutical Society of Singapore (PSS) Intermediate and Long-Term Care (ILTC) Pharmacists Workgroup Drug-Related Problem (DRP) Classification System.

No.	Main Categories	Subcategories	Remarks
1	Indication	(a) Drug use without indication	Patient is taking a drug without a valid medical indication
		(b) Untreated indication	Patient has medical problem that requires drug therapy but is not receiving medication for that indication Examples: • Conditions where patients are in need of prophylaxis or premedications but are not receiving them • Combination therapy required for synergistic effect but only one drug is used, e.g., antibiotics, chemotherapy
		(c) Therapeutic duplication	Inappropriate duplication of therapeutic group or active ingredients
2	Improper drug selection	(a) Contraindication	Patient has contraindications to the drug prescribed
		(b) More cost-effective drug available	Similarly effective alternative is available at a cheaper price
3	Dosage	(a) Drug dose is too low	Dosage regimen is not individualized for a specific patient, taking into consideration the appropriate drug, disease, and patient-specific information Examples: • Dosage regimen adjustment for patients with renal/hepatic impairment • Dose adjustment when switching between formulations, e.g., phenytoin tablets and syrup, controlled release to immediate release formulation
		(b) Drug dose is too high	
		(c) Dosage regimen not frequent enough	
		(d) Dosage regimen too frequent	
4	Duration	(a) Duration of treatment too short	Patient is prescribed a drug for a duration that is clinically inappropriate
		(b) Duration of treatment too long	
5	Drug form	(a) Inappropriate formulation/drug form	Formulation/drug form is not individualized to patient, e.g., sustained release medication given to patient with nasogastric tube
		(b) Inappropriate change in brand	Different preparations of the same drug may not be bioequivalent. For drugs that have a narrow therapeutic window, switch in brands can affect clinical outcomes, e.g., levothyroxine
6	Adverse drug reaction	(a) ADR (nonallergic)	Consistent with pharmacologic actions of the drug, occur commonly, are usually dose dependent, and are fairly predictable
		(b) ADR (allergic)	Allergic or idiosyncratic reactions that are independent of drug pharmacology. Rare, not dose related, and cannot be predicted. (To be differentiated from wrong drug used where patient has been experiencing allergic reactions for a period of time versus ADR, where patient is taking medication for first time and experiences allergic reaction)
7	Drug interaction	(a) Drug–drug interaction	E.g., Clarithromycin and simvastatin
		(b) Drug–food interaction	E.g., Dairy products and levothyroxine
		(c) Drug–lab interaction	E.g., Anaemia and HbA1c results
		(d) Drug–disease interaction	Clinically significant interaction between patient’s pre-existing medical conditions and drug prescribed
8	Others	(a) Lab monitoring	• Lack of routine lab monitoring, which is required for adjustment of drug dose, monitoring of side effects etc., e.g., random blood glucose, HbA1C, LFTs, lipid Panel, renal panel • Lab monitoring not performed at an interval that is clinically appropriate
		(b) RMR—related	E.g., illegibility, ambiguity, clarification of drug order, incomplete information, and lack of doctor’s signature at RMR, lack of nurses’ signature to sign on/off medication, lack of nurses’ signature to indicate medications served, inappropriate use of legends
		(c) Expired medication/inappropriate storage	• Patient given expired medication or medication that have not been stored properly • Patient misses dose as a result of expired or inappropriately stored medication
		(d) Nonavailability of medication	• Patient or family unable to afford medication, thus drug not made available • Drug prescribed is not available in Singapore or exemption medication or unable to be supplied to the institution • Inability to get the medication in time
		(e) Others—please state reason	Any unique/additional cases of DRPs that do not fall under any of the categories above • Please kindly specify the reason for putting it in this category and/or describe the DRP

**Table 2:** Participants’ Demographics.

Demographic Factors	N	%
Age		
21–30 years old	22	66.7
31–50 years old	11	33.3
Gender		
Male	9	27.3
Female	24	72.7
Working experience as a pharmacist		
0–5years	18	54.5
>5 years	15	45.5
Duration of involvement in ILTC sector		
0–3 years	22	66.7
>3 years	11	33.3
Type of ILTC institution		
Nursing home	27	81.8
Others *	6	18.2
Frequency of visit to the ILTC institution		
Less than once a month	5	15.2
Once in 1–4 weeks	21	63.6
More than once a week	7	21.2

* Three participants were from community hospitals, one from a hospice, and two unspecified.

**Table 3:** Users’ opinion on the usability of the classification system (adapted from AbuRuz et al.) [13].

	Positive Ratings ^a^	Neutral Ratings	Negative Ratings ^b^
	n (%)		
The DRP classification system allows me to choose the correct DRP category for the cases	29 (87.9)	3 (9.1)	1 (3.0)
The classification system is comprehensive	23 (69.7)	7 (21.2)	3 (9.1)
The classification system is easy to use	24 (72.7)	5 (15.2)	4 (12.1)
I am able to categorise the different types of DRPs efficiently by using the classification system	23 (69.7)	7 (21.2)	3 (9.1)
I will use such a classification system in future	23 (69.7)	6 (18.2)	4 (12.1)
In general, I am satisfied with the classification system	24 (72.7)	8 (24.3)	1 (3.0)

^a^ Positive rating includes “strongly agree” and “agree”; ^b^ Negative rating includes “strongly disagree” and “disagree”. In this study, none of the participants indicated “strongly disagree”.
